# Polymeric Nanovectors Incorporated with Ganciclovir and HSV-*tk* Encoding Plasmid for Gene-Directed Enzyme Prodrug Therapy

**DOI:** 10.3390/molecules26061759

**Published:** 2021-03-21

**Authors:** Alicia J. Sawdon, Jun Zhang, Sarah Peng, Esmael M. Alyami, Ching-An Peng

**Affiliations:** 1 Department of Chemical Engineering, Michigan Technological University, Houghton, MI 49931, USA; ajsawdon@gmail.com; 2 Department of Chemical and Biological Engineering, University of Idaho, Moscow, ID 83844, USA; junzhang0713@outlook.com (J.Z.); sarahpeng21@gmail.com (S.P.); alya7030@vandals.uidaho.edu (E.M.A.)

**Keywords:** polymeric micelles, ganciclovir, gene-directed enzyme prodrug therapy, prodrug, HT29 cells, HSV-*tk*

## Abstract

In the area of gene-directed enzyme prodrug therapy (GDEPT), using herpes simplex virus thymidine kinase (HSV-*tk*) paired with prodrug ganciclovir (GCV) for cancer treatment has been extensively studied. It is a process involved with two steps whereby the gene (HSV-*tk*) is first delivered to malignant cells. Afterward, non-toxic GCV is administered to that site and activated to cytotoxic ganciclovir triphosphate by HSV-*tk* enzyme expressed exogenously. In this study, we presented a one-step approach that both gene and prodrug were delivered at the same time by incorporating them with polymeric micellar nanovectors. GCV was employed as an initiator in the ring-opening polymerization of ε-caprolactone (ε-CL) to synthesize hydrophobic GCV-poly(caprolactone) (GCV–PCL), which was furthered grafted with hydrophilic chitosan to obtain amphiphilic polymer (GCV–PCL–chitosan) for the fabrication of self-assembled micellar nanoparticles. The synthesized amphiphilic polymer was characterized using Fourier transform infrared spectroscopy and proton nuclear magnetic resonance. Micellar prodrug nanoparticles were analyzed by dynamic light scattering, zeta potential, critical micelle concentration, and transmission electron microscopy. Polymeric prodrug micelles with optimal features incorporated with HSV-*tk* encoding plasmids were cultivated with HT29 colorectal cancer cells and anticancer effectiveness was determined. Our results showed that prodrug GCV and HSV-*tk* cDNA encoded plasmid incorporated in GCV–PCL–chitosan polymeric nanocarriers could be delivered in a one-step manner to HT-29 cells and triggered high cytotoxicity.

## 1. Introduction

To facilitate tumors susceptible to enzyme-prodrug cancer therapy, prodrug-activating exogenous enzymes can be delivered to tumor cells using genes. This approach is so-called gene-directed enzyme prodrug therapy (GDEPT) [1]. It is a two-step treatment for cancer therapy. Enzymes are delivered to and expressed in target cells where they can activate subsequently administered non-toxic prodrugs to cytotoxic drugs. In the first step, a gene expressing the enzyme is delivered. In the second step, a prodrug is administered that can be activated to a toxic drug by the enzyme that has been expressed in the tumor. So far, the delivery of prodrug and gene was conducted via separate routes [2,3]. The widely used GDEPT system is herpes simplex virus thymidine kinase (HSV-*tk*) in combination with ganciclovir prodrug [4,5]. Ganciclovir (GCV) is also a well-known antiviral agent [6,7,8]. The HSV-*tk*/GCV system has been employed to catalyze GCV into its cytotoxic form [i.e., ganciclovir triphosphate (GCV-TP)] by exogenously expressed HSV-*tk* enzyme for the treatment of brain tumors [9,10], colorectal tumors [11,12], and head/neck tumors [13,14]. Published accounts have shown that HSV-*tk*/GCV exhibits not only cytotoxicity but also a bystander effect that augments the anticancer effectiveness [5,15,16]. That being said, significant therapeutic benefit has been limited by the delivery efficiency to tumor cells. The studies of GDEPT have been focused on using viral vectors to deliver activating genes to malignant cells [17,18]. However, viruses have the risk of rendering tumorigenicity and immunogenicity [19]. Safety concerns involved with viral vectors have triggered the development of nonviral vectors for cancer gene therapy. In particular, polymer micellar nanoparticle formulations have gained attention due to their widespread use as drug delivery vehicles [20,21,22,23].

In this study, we intend to develop a single polymeric micellar carrier that contains both prodrug GCV and its activating HSV-*tk* gene for establishing a one-step approach for GDEPT. The polymeric nanocarrier is composed of a hydrophobic inner core and a hydrophilic outer shell. Poly(ε-caprolactone) (PCL) having been widely used as the core-forming hydrophobic segment of nanoparticles is selected as the model polymer. PCL is linear resorbable aliphatic polyester. It has been frequently explored as implantable carriers for drug delivery systems due to the biocompatible nature of the degradation products [24]. Moreover, PCL is currently approved by the FDA for use in humans. PCL is commonly synthesized by ring-opening polymerization of ε-caprolactone using alcohol (R-OH) as an initiator and stannous (II) octoate (Sn(Oct)_2_) as a catalyst [25,26]. Prodrug GCV possessing hydroxyl groups was employed as the initiator to synthesize GCV–PCL by ring-opening polymerization of ε-caprolactone [27]. The reported liphophilic GCV–PCL were further grafted with hydrophilic biodegradable chitosan which has been widely accepted as an efficient vector for nonviral gene delivery by forming complexes with DNA plasmids [28,29].

**Figure 1:**
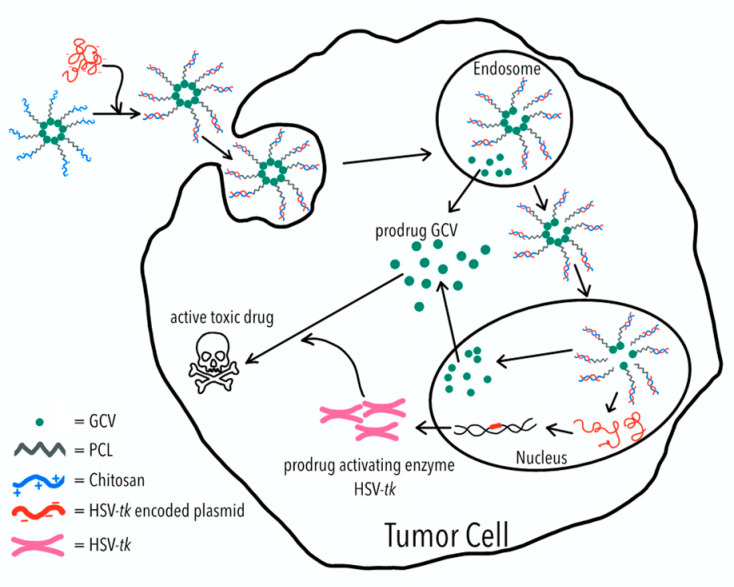
Polymeric micelles of ganciclovir–poly(caprolactone) (GCV–PCL)-chitosan are fabricated due to the hydrophobicity of PCL and cationic hydrophilicity of chitosan. The GCV–PCL–chitosan/HSV-*tk* complex is assembled through ionic interactions. For each tumor cell, the GCV–PCL–chitosan/HSV-*tk* complex is first ingested into the endosome and then transported into the nucleus which allows HSV-*tk* gene to be expressed into prodrug activating enzyme which converts prodrug GCV into an active toxic drug (i.e., GCV–TP) for tumor cell destruction.

To evaluate the anticancer efficacy of the self-assembled polymeric micelles formed from the synthesized amphiphilic polymer, HT29/HSV-*tk* cells which expressed HSV-*tk* gene encoding plasmid were treated with synthesized polymeric prodrug micelles. Moreover, the positive charge endowed on the micelles by chitosan was used to attract negatively charged HSV-*tk* gene encoding plasmid onto the polymeric prodrug micelles. The GCV-embedded polymeric micelles formed polyplexes with HSV-*tk* plasmids were harnessed for one-step GDEPT cancer therapy to parental HT29 colorectal cells, as illustrated in Figure 1. Our results indicate that GCV–PCL–chitosan/HSV-*tk* nanovectors are potent carriers for one-step GDEPT.

## 2. Results and Discussion

### 2.1. Synthesis and Characterization of Amphiphilic Prodrug Polymers

**Figure 2:**
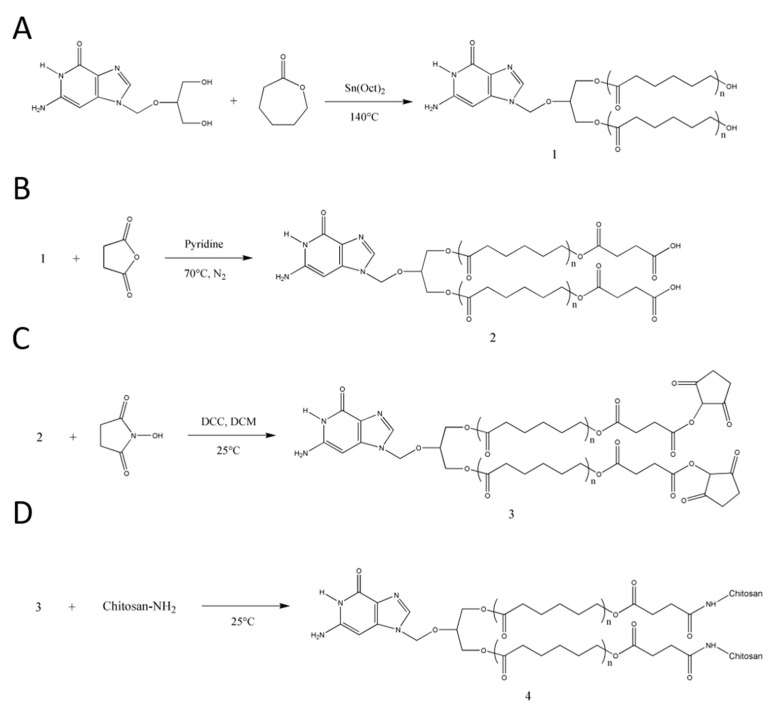
Synthetic scheme of (**A**) GCV–PCL, (**B**) GCV–PCL–COOH, (**C**) GCV–PCL–NHS, and (**D**) GCV–PCL–chitosan.

**Figure 3:**
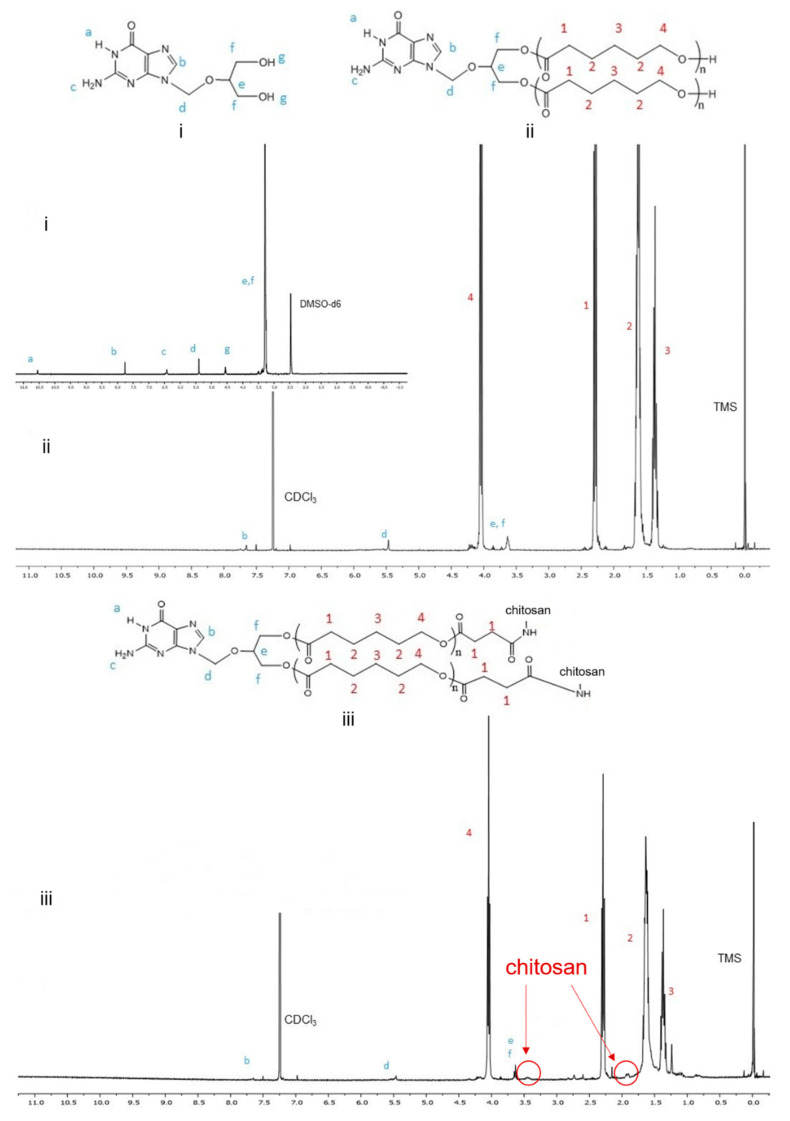
Proton NMR spectra of (**i**) GCV, (**ii**) GCV–PCL, and (**iii**) GCV–PCL–chitosan.

GCV–PCL was synthesized using ring-opening polymerization of ε-caprolactone via the hydroxyl groups on GCV (Figure 2A). The proton nuclear magnetic resonance (NMR) spectra of prodrug GCV and GCV–PCL are shown in Figure 3(i) and (ii), respectively. Chemical shifts at δ = 2.27 (1-CH_2_), 1.62 (2-CH_2_), 1.37 (3-CH_2_), and 4.04 (4-CH_2_) ppm correspond with the backbone chain of a polycaprolactone polymer. Peaks at δ = 3.63 (e, f-CH_2_), 5.47 (d-CH_2_) and 7.76 (b-CH) are corresponding to the protons in prodrug GCV. The synthesis of GCV–PCL is confirmed by the characteristic resonances revealed in the obtained polycaprolactone incorporated with prodrug GCV.

GCV–PCL was further grafted with chitosan as shown in Figure 2B–D. Proton NMR analysis (see Figure 3iii) confirms the successful grafting of chitosan to GCV–PCL hydrophobic polymer. As shown in Figure 2D, chitosan grafted to GCV–PCL is made via amide linkage. The peak at δ = 1.79 (l-NH_2_) from a singlet to a multiplet in Figure 3iii confirms conjugation of chitosan to GCV–PCL. Moreover, the peaks from the protons on C_3_–C_6_ of chitosan can be seen from δ = 3.28–3.85.

Moreover, the gel permeation chromatography (GPC) data shown in Table 1 confirmed the formation of amphiphilic copolymer GCV–PCL–chitosan. GCV–PCL hydrophobic polymer had an observed number-average molecular weight (M_n_) of 11.5 kDa which increased to 17.2 kDa after the addition of chitosan. This corresponds well with the addition of chitosan which had an average molecular weight of 5 kDa. Furthermore, the polydispersity index (PDI) of both GCV–PCL and GCV–PCL–chitosan polymer was low at 1.13 and 1.18, respectively which indicates that the polymer chains are approaching a uniform chain length. The molecular weights of GCV–PCL and GCV–PCL–chitosan were found via GPC calibrated by polystyrene standards. The molar ratio of GCV–PCL to chitosan used for the synthesis was calculated to be 1:5.

**Figure 4:**
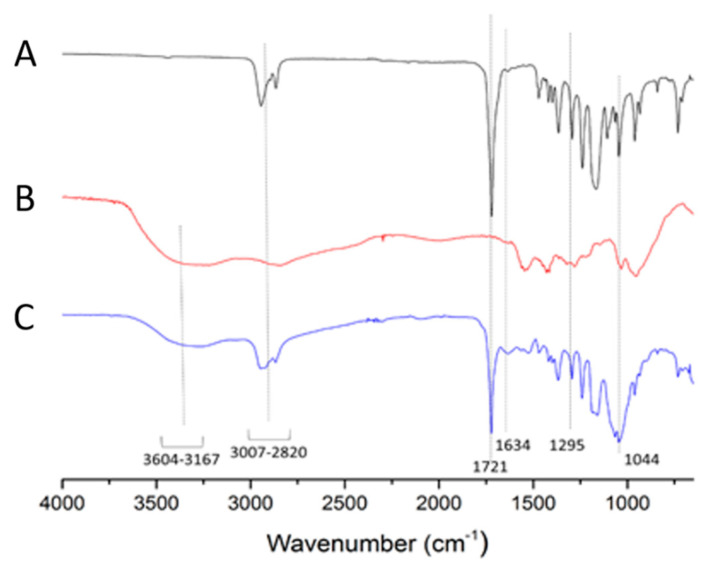
FTIR spectra of (**A**) GCV–PCL, (**B**) chitosan, and (**C**) GCV–PCL–chitosan.

Figure 4 reveals the Fourier transform infrared (FTIR) spectra of GCV–PCL (A), chitosan (B), and GCV–PCL–chitosan (C). OH stretching from 3604–3167 cm^−1^ as well as peaks at 1634 cm^−1^ and 1295 cm^−1^ corresponds to N-H bending vibrations of primary and secondary amine endowed by chitosan and GCV, respectively. The above-mentioned peaks and the carbonyl absorption at 1721 cm^−1^ associated with PCL and a peak at 1044 cm^−1^ (C–O–C) shown in each spectrum are observed in GCV–PCL–chitosan. The FTIR results are consistent with the outcomes from proton NMR and assure the successful synthesis of GCV–PCL–chitosan.

### 2.2. Characterization of GCV-Tagged Polymeric Micelles and Their Complexes with HSV-tk

**Figure 5:**
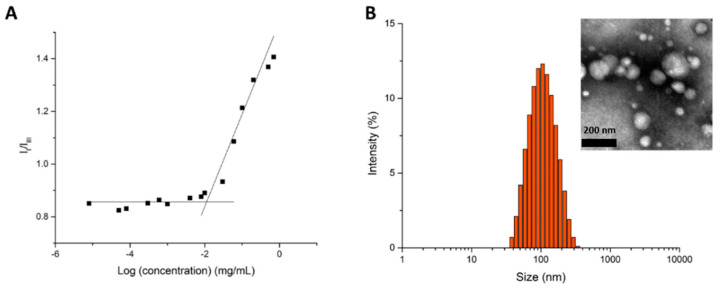
(**A**) Plot of the intensity ratio (I_338_/I_329_) versus concentration of GCV–PCL–chitosan polymeric micelles and (**B**) size distribution of GCV–PCL–chitosan polymeric micelles. Inset represents the transmission electron microscopy (TEM) image.

Polymeric micelles of GCV–PCL–chitosan were fabricated by the solvent evaporation method, using GCV–PCL as the hydrophobic core section and chitosan as the cationic and hydrophilic corona section. Pyrene was utilized as a hydrophobic fluorescent probe to determine the critical micelle concentration (CMC) of GCV–PCL–chitosan polymeric micelles. Pyrene could partition into the hydrophobic core (i.e., GCV–PCL segment) microdomains and alter the intensities of the first and third bands in the pyrene fluorescence spectrum [30]. A low CMC is an indication that the micellar solution is stable at high dilutions. For GCV–PCL–chitosan polymeric micelles, the shift of the first and third bands was observed at I_338_/I_329_. The critical micelle concentration was calculated to be 1.12 × 10^−2^ mg/mL (Figure 5A). To determine the prodrug GCV loading percentage in the formed GCV–PCL–chitosan polymeric micellar solution, the absorbance at 420 nm (maximum peak of GCV) of GCV–PCL–chitosan polymeric micelles was measured at t = 0 h and t = 72 h. The standard calibration curve of GCV absorbance (at 420 nm) versus GCV concentration ranging from 0.002 to 1.0 mg/mL was employed to determine the amount of GCV in the polymeric prodrug micelles. The percentage of prodrug GCV incorporated in GCV–PCL–chitosan polymeric micelles was calculated to be 4.8%.

As shown in Figure 5B, the morphology and size of GCV–PCL–chitosan polymeric micelles were determined by TEM and dynamic light scattering (DLS) analysis. The average size of polymeric micelles as determined by DLS was 93.4 nm with a zeta potential of 38.5 mV. This positive charge is attributed to chitosan employed as the hydrophilic portion on the polymeric prodrug micelle carriers. TEM analysis, shown in the inset of Figure 5B, illustrates the spherical morphology of the polymeric micelles. The size distribution GCV–PCL–chitosan polymeric micelles obtained from DLS analysis is consistent with the one analyzed from the TEM images. 

After the preparation of GCV–PCL–chitosan polymeric micelles, various amounts of HSV-*tk* gene encoding plasmids (1.5, 2.0, 3.0, and 4.5 µg) were mixed with 1 mL of polymeric micelle solution. Due to electrostatic interaction, the negatively charged plasmids were able to form a complex with the positively charged chitosan employed as the hydrophilic section in polymeric micelles. Size and charge measured by DLS and zeta potential were listed in Table 2. The DNA plasmid was incubated for 30 min to form complexes with GCV–PCL–chitosan polymeric micelles. The size of formed GCV–PCL–chitosan/HSV-*tk* nanovectors was increased up to 128.4 nm with the addition of 4.5 µg of HSV-*tk* gene encoding plasmids. Accordingly, the charge gradually decreased from 38.5 to 28.2 mV after cationic polymeric micelles complexes with more (up to 4.5 µg) negatively charged HSV-*tk* DNA plasmid.

### 2.3. Release of GCV from Polymeric Micelles

**Figure 6:**
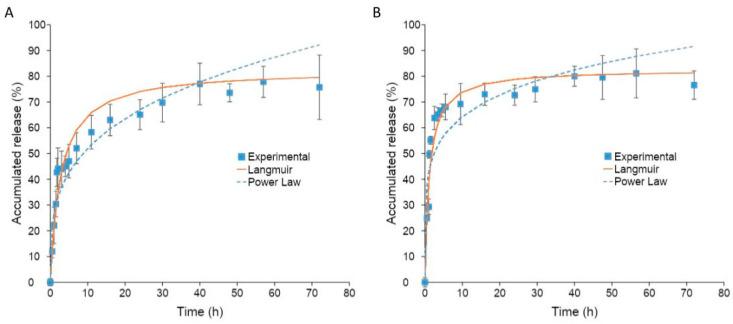
(**A**) GCV drug release profile from GCV–PCL–chitosan polymeric micelles in PBS at 37 °C (mean ± SD, *n* = 3). (**B**) GCV drug release profile from GCV–PCL–chitosan polymeric micelles in PBS at 37 °C with esterase (1.5 units/mL) (mean ± SD, *n* = 3).

Esterase with the enzyme activity of 1.5 units/mL was used to mimic the cellular environment [31]. As illustrated in Figure 6A, it took 48 h without esterase for GCV to reach a maximum release of 77%. In contrast, the release of GCV with esterase was much faster and reaching a maximum release of 81% within 30 h (Figure 6B). Moreover, it took up to 2 h before a substantial difference in the release rate was observed. This is because of the time required for esterase diffusing into the hydrophobic core of polymeric micelles for the cleavage of an ester bond between GCV and PCL.

The release profile of GCV from GCV–PCL–chitosan polymeric micelles was fitted with the Langmuir model and power-law model. As illustrated in Figure 6A,B, the power-law model was not a good fit for the release of GCV from polymeric micelles. The exponent n of the power-law model was equal to 0.18 and 0.29 for polymeric micelles with and without esterase, respectively. The mechanism of drug release was not solely by diffusion [32]. The release of GCV without esterase occurred by hydrolysis of the ester bond between GCV and PCL. The release rate was increased by the addition of esterase to polymeric micelles due to enhanced hydrolysis of the ester bond attributed to esterase. Since GCV release happened by reactive diffusion, the Langmuir model was therefore chosen for fitting the experimental data. As shown in Figure 6A,B, the release of GCV from polymeric prodrug micelles with and without esterase using the Langmuir model fits the experimental data better. Moreover, the dissociation constant for the release of GCV from polymeric prodrug micelles, was calculated to be 1.18 and 2.79 with and without esterase, respectively.

### 2.4. Characterization of HT29/HSV-tk Cells

**Figure 7:**
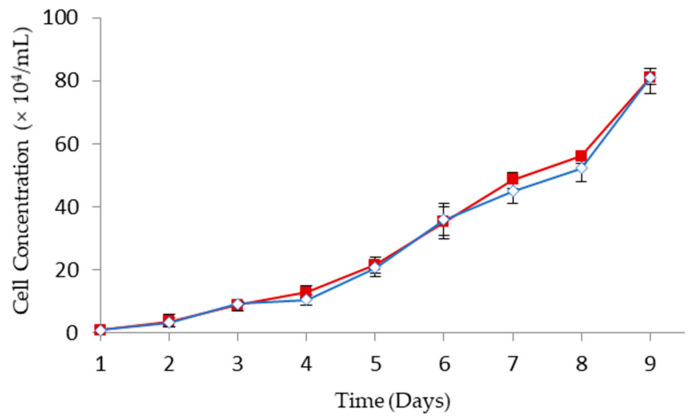
Cell growth kinetics of HT29 (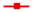) and HT29/HSV-*tk* (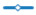) cells (mean ± SD, *n* = 3).

To investigate the efficacy of GCV for GDEPT, HT29 colorectal cancer cells were transfected with HSV-*tk* gene encoding plasmid complexed with MPEG–PCL–chitosan polymeric micelles. As shown in Figure 7, the growth kinetics of HSV-*tk* gene transfected HT29 (i.e., HT29/HSV-*tk*) cells is similar to the one obtained for parental HT29 cells. This indicates that the antibody selection of a stably transfected cell line was not altered in the growth profile.

**Figure 8:**
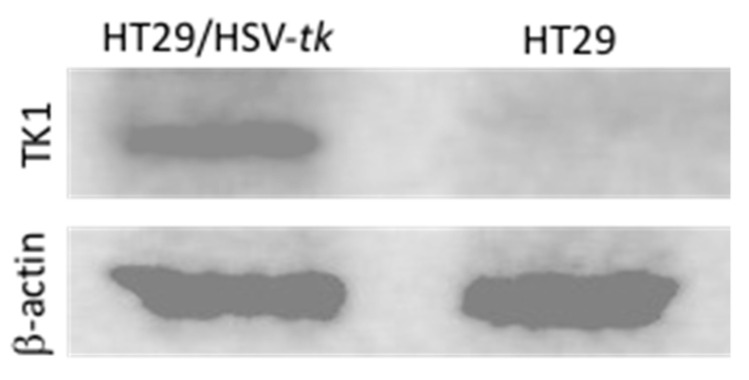
Western blot analysis of HT29/HSV-*tk* cells performed to detect and verify expression of HSV-*tk* in transfected HT29 cells. *β*-actin was used as a housekeeping gene.

A Western blot analysis was conducted to detect and verify the expression of HSV-*tk* in HT29 cells transfected with HSV-*tk* gene using MPEG–PCL–chitosan polymeric micelles. To demonstrate the robust expression of HSV-*tk* in the cell line, total proteins were extracted and analyzed for HSV-*tk* expression via Western blot. As shown in Figure 8, a strong band corresponding to HSV-*tk* (~50 kD) is observed in protein extracts from transfected HT29/HSV-*tk* cells (lane 1), whereas the untransfected parental HT29 cells as negative control display no HSV-*tk* expression (lane 2). The expression of *β*-actin used as internal control detected at ~43 kD reveals a similar level for the protein extracted from transfected HT29/HSV-*tk* cells and untransfected parental HT29 cells. These results confirmed that MPEG–PCL–chitosan/HSV-*tk* nanovectors could efficiently induce high levels of HSV-*tk* expression in HT29 cells. It is expectable that GCV–PCL–chitosan/HSV-*tk* nanocomplexes could produce significant expression of HSV-*tk* enzyme, thereby resulting in the death of transfected HT29 cells via converting prodrug GCV into toxic GCV–TP in the presence of exogenously expressed HSV-*tk* enzyme.

### 2.5. Cytotoxicity Studies

**Figure 9:**
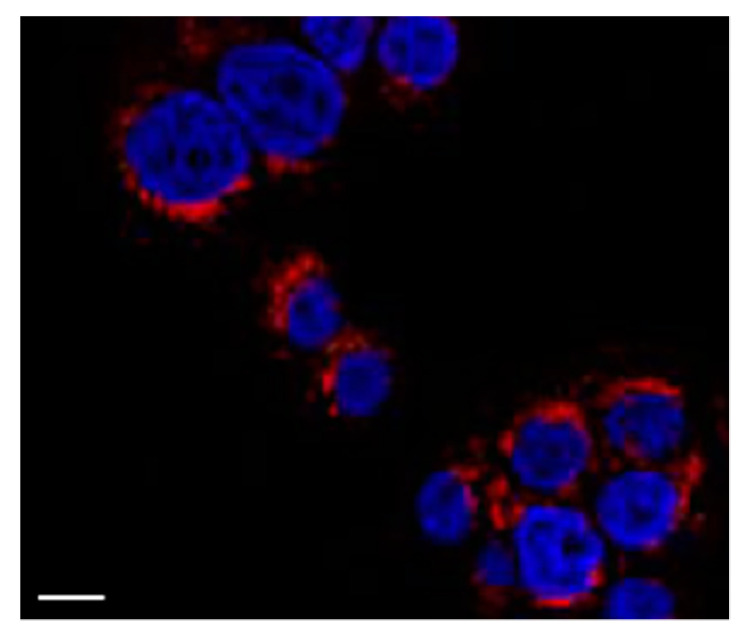
Fluorescent image of HT29 cells treated with GCV–PCL–chitosan polymeric micelles encapsulated with Nile Red for 6 h. The blue fluorescence is 4′,6-diamidino-2-phenylindole (DAPI) employed to stain cell nucleus and the red fluorescence is Nile Red. Scale bar = 10 μm.

To explore the interaction between polymeric micelles and HT29 cells, the cellular uptake of GCV–PCL–chitosan polymeric micelles loaded with hydrophobic Nile Red fluorescent dye was used. As shown in Figure 9, endocytic uptake of polymeric micelles was evidenced by the red fluorescence of Nile Red incorporated in the hydrophobic core domain of the polymeric micellar nanoparticles dispersed in the cytosol. This observation is consistent with the reported endocytosis mechanism for polymeric micelles [33,34,35,36].

**Figure 10:**
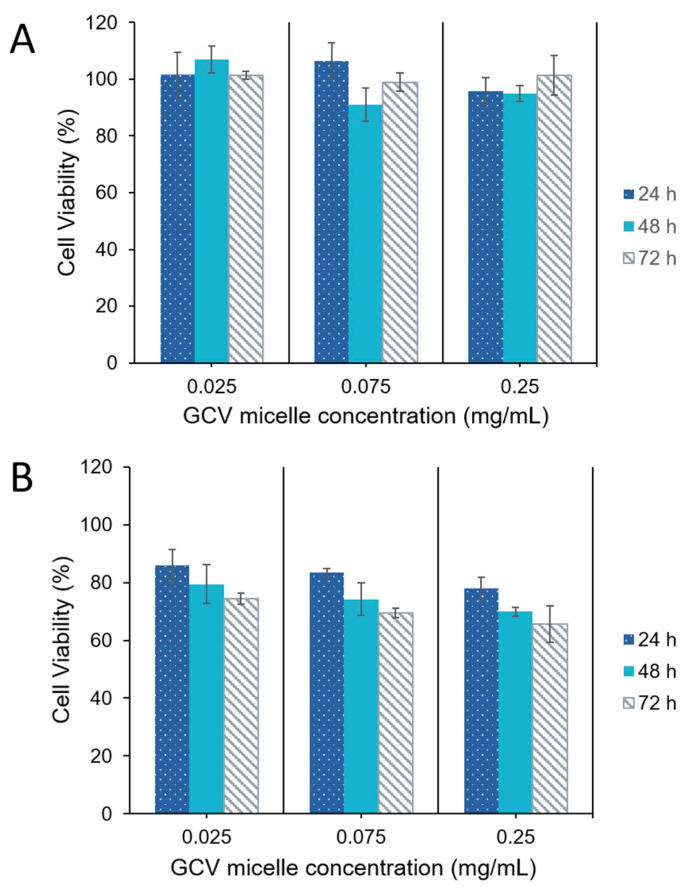
Viability of (**A**) parental HT29 cells, and (**B**) HT29/HSV-*tk* cells post-treatment with GCV–PCL–chitosan polymeric micelles (mean ± SD, *n* = 3).

The stable transfected HT29/HSV-*tk* cells and parental HT29 cells were treated with GCV-incorporated polymeric micelles. Cytotoxicity of GCV–PCL–chitosan polymeric micelles with concentrations of ranged from 0.025 to 0.25 mg/mL for 24, 48 and 72 h respectively was examined by the 3-(4,5-dimethylthiazol-2-yl)-2,5-diphenyltetrazolium bromide (MTT) assay. Since parental HT29 cells do not have any endogenous HSV-*tk* enzyme to convert GCV into its toxic form (i.e., GCV–TP), cell viability was not affected (Figure 10A). In contrast, HT29/HSV-*tk* cells which had upregulated HSV-*tk* gene expression revealed a 35% increase in cell death at a dosage of 250 µg/mL post 72 h treatment (Figure 10B). These outcomes confirmed that prodrug GCV could be converted into GCV–TP by HSV-*tk* enzyme expressed in HT29 cells for a two-step GDEPT.

**Figure 11:**
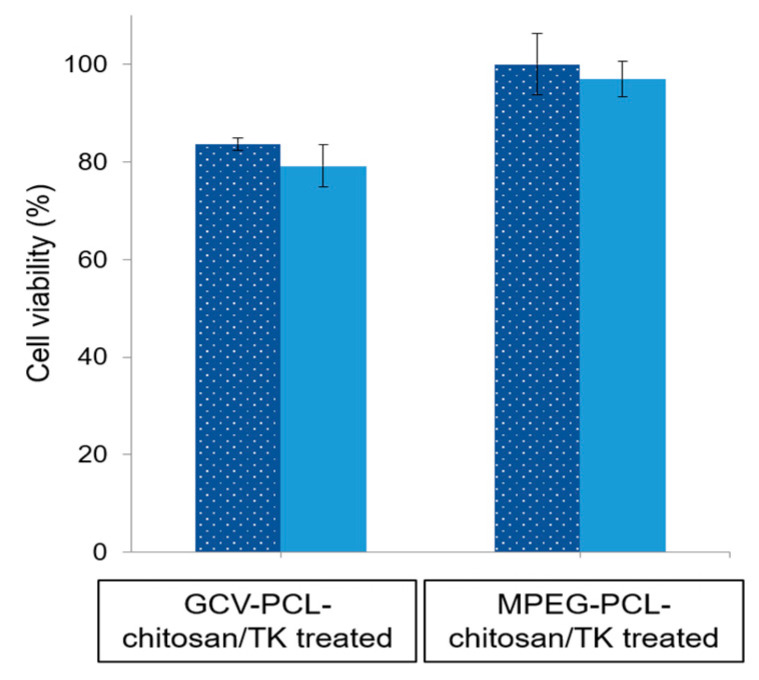
HT29 cell viability after challenged with GCV–PCL–chitosan/HSV-*tk* nanovectors and MPEG–PCL–chitosan/HSV-*tk* nanovectors, respectively for 3 (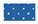) and 5 (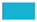) days (mean ± SD, *n* = 3).

To study the feasibility of a one-step GDEPT approach, HSV-*tk* gene encoding plasmids (1.5 µg) were complexed onto GCV–PCL–chitosan polymeric micelles via electrostatic interaction. The expression of HSV-*tk* gene and ensuing cell toxicity was examined for three and five days. As demonstrated in Figure 11, the level of HSV-*tk* enzyme expressed in HT29 cells after three days could lead to ~20% cell death. An additional two days resulted in no significant increase in cell toxicity. The internalization of GCV–PCL–chitosan/HSV-*tk* nanovectors in endosome/lysosome vesicles led to the release of GCV prodrug (due to the cleavage of ester bond) and nanovectors. The latter one then further transported into the nucleus and resulted in the expression of GCV-activating HSV-*tk* enzyme that eventually converted GCV into toxic GCV–TP for malignant cell destruction (see Figure 1 for illustration). On the contrary, when the HSV-*tk* gene encoding plasmid was complexed onto control polymeric micelles (MPEG–PCL–chitosan), cell toxicity was not detected. This is because the MPEG-incorporated polymeric micelles could not be converted into toxic form by HSV-*tk* enzyme like their counterparts (i.e., GCV-incorporated polymeric micelles) did to HT29 cells.

**Figure 12:**
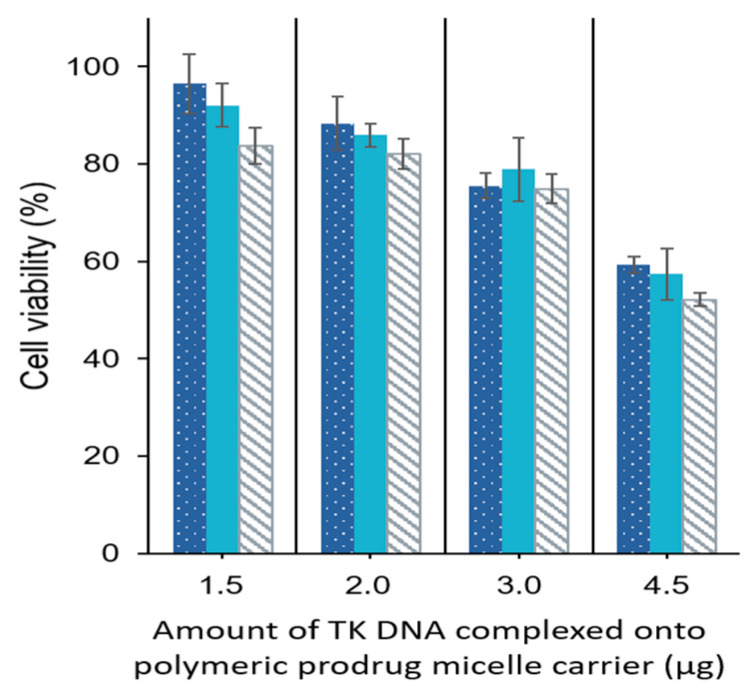
One-step approach for the delivery of gene and prodrug. HT29 cells were treated with 25 (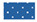), 75 (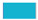), and 250 (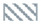) µg/mL GCV–PCL–chitosan complexed with various amounts (1.5, 2.0, 3.0, and 4.5 µg) HSV-*tk* gene encoding plasmid for 3 days (mean ± SD, *n* = 3).

HT29 cells were further incubated with three different concentrations (25, 75, and 250 µg/mL) of GCV–PCL–chitosan/HSV-*tk* nanovectors complexed with various amounts (1.5, 2.0, 3.0, and 4.5 µg) of HSV-*tk* gene encoding plasmid. As indicated in Figure 12, increasing the amount of plasmid, more GCV was converted to its cytotoxic form (i.e., GCV–TP) resulting in more cell death. Cell viability decreased to ~52% with polymeric prodrug micelle concentration of 250 µg·mL^−1^ and 4.5 µg HSV-*tk* gene encoding plasmid. It seems that 4.5 µg of plasmids is the upper limit for complexation with polymeric prodrug micelles. Since the maximum cytotoxicity of GCV–PCL–chitosan/HSV-*tk* nanovectors obtained was not significantly high (about 50%), the gene transfection efficiency of GCV–PCL–chitosan/HSV-*tk* nanovectors probably was not very high (i.e., only a sub-population was transfected). This could be due to the low endosomal escape of the nanovectors. It is speculated that anticancer effectiveness could be enhanced by changing chitosan to polyethylenimine (PEI) which could improve endosomal escape [37,38,39]. It should be noted that the hydrophobic core of polymeric prodrug micelles was not loaded with any other anticancer drugs. It is foreseeable that, with the encapsulation of hydrophobic chemotherapy drug (e.g., SN-38) into GCV-incorporated polymeric micelles, HT29 cells will have more death than the ones shown in Figure 12 due to the combination effect of both GCV and chemotherapy drug on HT29 cells expressing HSV-*tk* enzyme [40].

## 3. Materials and Methods

### 3.1. Materials

HT29 cells was purchased from ATCC (Manassas, VA, USA. Dulbecco’s modified Eagles’ medium (DMEM) was purchased from Corning Cellgro (Manassas, VA, USA). Fetal bovine serum (FBS), mouse IgG_1_ horseradish peroxidase (HRP)-conjugated antibody, and MTT cell proliferation/viability assay kits were all purchased from R&D Systems (Minneapolis, MN). CDCl_3_ with 1% tetramethylsilane (TMS), Sn(Oct)_2_, pyridine, ε-caprolactone, magnesium sulfate, sodium chloride, succinic anhydride, hydrochloric acid (HCl), dimethyl sulfoxide (DMSO), deuterated dimethyl sulfoxide (DMSO-*d*_6_), dichloromethane (DCM), *N*-hydroxysuccinimide (NHS), *N*,*N*’-dicyclohexyl carbodiimide (DCC), methanol, tetrahydrofuran (THF), acetone, 2-propanol, diethyl ether, toluene, hexane, methoxy poly(ethylene glycol) (MPEG, MW = 350), chitosan oligosaccharide lactate (MW = 5000), pyrene, penicillin-streptomycin, G418 disulfate, esterase from porcine liver, Nile Red, and 4′,6-diamidino-2-phenylindole (DAPI) were all purchased from Sigma-Aldrich (St. Louis, MO, USA). GCV was purchased from TCI (Tokyo, Japan). RIPA lysis buffer, Halt™ protease inhibitor, phosphate-buffered saline (PBS), enhanced chemiluminescence (ECL) substrate, Tris buffer saline (TBS), Tween-20, and mouse TK1 monoclonal antibody (clone 3B3 E11) were purchased from Thermo Fisher Scientific (Waltham, MA, USA). Bovine serum albumin (BSA), laemmli sample buffer, and 2-mercaptoethanol were obtained from Bio-Rad (Hercules, CA, USA). Polyvinylidene difluoride (PVDF) membrane was purchased from Pall Life Science (Port Washington, NY, USA). Mouse *β*-actin monoclonal antibody (sc-47778) and mouse IgG_K_ binding protein-HRP monoclonal antibody (sc-516102) were purchased from Santa Cruz Biotechnology (Dallas, TX, USA).

### 3.2. Synthesis of GCV-Incorporated and MPEG-Incorporated Amphiphilic Polymers

The synthesis of GCV–PCL–chitosan and MPEG_350_–PCL–chitosan amphiphilic polymers in this study was previously reported [27]. In brief, 50 mg of GCV was mixed with 2.25 mL of ε-caprolactone (ε-CL) for 5 min at room temperature, followed by the addition of Sn(Oct)_2_ (0.5 wt% of ε-CL). The system was nitrogen-purged and immersed in an oil bath at 140 °C for 24 h. The final product was then vacuum dried by rotary evaporation at 40 °C. 0.5 mmol of GCV–PCL and 1 mmol of succinic anhydride were dissolved in toluene, followed by the addition of 1 mmol pyridine. The mixture was allowed to react at 70 °C for 48 h under nitrogen purging. The carboxylated GCV–PCL was recovered by precipitation in cold hexane and then vacuum dried by rotary evaporation at 40 °C. Then, 0.54 mmol of GCV–PCL–COOH and 2.7 mmol of NHS were weighed and added to 15 mL DCM, and then 2.7 mmol of DCC was added. The mixture was allowed to react at room temperature for 24 h under nitrogen purging. The solvent was removed by rotary evaporation at 40 °C to obtain GCV–PCL–NHS. A chitosan solution was prepared by dissolving 20 mg chitosan oligosaccharide lactate in 25 mL deionized water. 10 mg of GCV–PCL–NHS was then dissolved in 5 mL acetone and slowly added to this chitosan solution. The mixture was reacted for 24 h under nitrogen purging. The reaction mixture was vacuum dried to remove acetone and then lyophilized. The amphiphilic polymer was finally dissolved in DCM and dialyzed (Molecular weight cutoff = 6–8 kD, Spectra/Por, New Brunswick, NJ, USA) against pure DCM to remove unreacted chitosan. GCV–PCL–chitosan was recovered by rotary evaporation at 40 °C. In control polymeric micelles, MPEG_350_–PCL was the hydrophobic core segment and chitosan was the hydrophilic segment. The reason for selecting MPEG_350_ as the initiator for control studies was due to the fact that MPEG_350_ has a molecular weight close to GCV’s (MW = 255.23 g/mol).

### 3.3. Characterization of GCV–PCL and GCV–PCL–Chitosan

Proton NMR spectra were obtained from a 400 MHz instrument (Varian Unity/Inova, Sparta, NJ, USA). Fourier transform infrared (FTIR) spectra were obtained from a FTIR-4200 spectrometer (Jasco, Tokyo, Japan) by loading a small amount of polymer-dissolved THF onto a silicon wafer and forming a film after THF evaporation. Gel permeation chromatography (GPC) analyses were performed on a Waters 1525 binary high performance liquid chromatography (HPLC) pump equipped with a Waters 2414 refractive index detector (Milford, MA, USA). Waters styragel HR 3 (MW = 500–30,000) and HR 4E (MW = 50–100,000) columns were equipped. Molecular weight calibration was performed with polystyrene standards that covered a MW range of 400–4.3 × 10^4^. GPC analyses were performed in THF at a flow rate of 1 mL·min^−1^ with an injected volume of 50 µL.

### 3.4. Preparation of Polymeric Prodrug Micelles

GCV–PCL–chitosan micelles and control MPEG–PCL–chitosan micelles were formed similarly. Briefly, 10 mg of GCV-tagged amphiphilic polymer (or MPEG-tagged amphiphilic polymer) was dissolved in 2 mL acetone. The solution was then added dropwise to 10 mL deionized water under sonication. Acetone was removed by rotary evaporation and the solution was collected and filtered through a 0.45 µm filter.

### 3.5. Determination of Critical Micelle Concentration

The critical micelle concentration (CMC) was estimated by using fluorescent pyrene [41]. Briefly, 1 mg·mL^−1^ of polymeric prodrug micelle was formed and diluted with various amounts of deionized water to obtain micellar concentrations ranging from 5 × 10^−7^ to 1 mg/mL. Pyrene in acetone was then added to the above micelle solutions to reach a final concentration of 6.0 × 10^−7^ mg/mL. The solutions were then equilibrated at room temperature for 8 h. Fluorescent spectra were determined by a microplate reader (SpectraMax M2e, Molecular Devices, Sunnyvale, CA, USA) with an excitation wavelength of 334 nm.

### 3.6. Size and Morphology of Polymeric Prodrug Micelles

The hydrodynamic size of polymeric micelles was detected by a DLS instrument (Zetasizer Nano ZS, Malvern Instruments, Westborough, MA, USA). The zeta potential of the polymeric micelles was determined with a zeta potential analyzer (Zetasizer Nano ZS). TEM image of polymeric micelles was analyzed by a JEM-4000FX (JEOL, Tokyo, Japan) at 80 kV. The TEM samples were prepared by adding 10 µL of polymeric micellar solution (1 mg/mL) onto a Formvar grid for 5 min. The samples were negatively stained with 10 µL of 2 wt% phosphotungstic acid solution.

### 3.7. Drug Release Kinetics

1 mg·mL^−1^ of polymeric prodrug micelles were prepared in phosphate-buffered saline (PBS) (1 M, pH 7.4) at 25 °C. 2 mL of the micelle solution was placed in a dialysis tube (Float-A-Lyzer, Spectra/Por, New Brunswick, NJ, USA) with an MWCO of 3.5–5 kD. The dialysis bag was then immersed in 50 mL of PBS at 37 °C with and without esterase (3 units/2 mL). 5 µL of the sample was removed and replaced with fresh PBS at various time points to analyze the amount of GCV released by a microplate reader (SpectraMax M2e, Molecular Devices, Sunnyvale, CA, USA) at 254 nm. All experiments were carried out in triplicate.

### 3.8. Establishing HSV-tk-Expressed HT29 Cells

The recombinant pcDNA3-HSV-*tk* plasmid, generously provided by Dr. Kang Fang (National Taiwan Normal University, Taipei, Taiwan), was constructed by ligating the HSV-*tk* gene into pcDNA3 mammalian vector (Invitrogen, Carlsbad, CA, USA) with cytomegalovirus promotor [42]. The pcDNA3-HSV-*tk* plasmid was transformed into DH5α competent cells and the plasmids were purified using the Qiagen Maxiprep kit (Valencia, CA, USA). The purity and concentration of isolated plasmid DNA were determined by measuring absorption at 260 and 280 nm using UV spectrophotometry. To construct HSV-*tk* expressed cells, human colorectal HT29 cells (HTB-38; ATCC, Manassas, VA, USA) were inoculated at a cell density of 6 × 10^5^ cells cm^−2^, and incubated with 2.5 µL HSV-*tk* plasmid DNA and 5 µL MPEG–PCL–chitosan polymeric micelles. After 48 h, cells were trypsinized and suspended in a fresh medium containing 400 µg·mL^−1^ of antibiotic G418 disulfate. Cells were selected for several weeks to obtain a stable HSV-*tk* expressing an HT29 cell line (termed HT29/HSV-*tk*). The cell growth curves of HT29 and HT29/HSV-*tk* were determined over 9-day cultivation by counting cell numbers using a hemocytometer.

### 3.9. Detection of HSV-tk Protein Expression

To determine the level and size of HSV-*tk* in HT29 cells that were transfected, a Western blot analysis was performed using a monoclonal antibody against HSV-*tk*. Both HT29/HSV-*tk* and parental HT29 cells were harvested by trypsinization and washed with 1× PBS. To extract the proteins, cell pellets were resuspended in RIPA lysis buffer supplemented with 1× Halt protease inhibitor and sonicated on ice. Soluble proteins were isolated by centrifugation at 13,000× *g* at 4 °C for 20 min. Subsequently, proteins were mixed with an equal volume of Laemmli sample buffer supplemented with 5% (*v*/*v*) of 2-mercaptoethanol before heating to 95 °C for 5 min. The mixture was loaded onto 12% sodium dodecyl sulphate-polyacrylamide gel electrophoresis (SDS-PAGE) that performed at 200 V for 45 min and electroblotted onto PVDF membrane using a Trans-Blot SD semi-dry transfer cell (Bio-Rad, Hercules, CA, USA) for 1 h at 20 V. Membrane was blocked for 1 h at room temperature with 5% BSA in TBS-T (1 × TBS buffer with 0.1% Tween-20) under gently shaking condition. Then, the membrane was incubated with mouse TK1 monoclonal antibody (1:500 dilution) in blocking buffer with gentle shaking overnight at 4 °C. Afterward, the membrane was washed three times with TBS-T for 5 min each at room temperature before incubating with mouse IgG_1_ horseradish peroxidase (HRP)-conjugated antibody (1:1000 dilution) for 2 h at room temperature. Following three times washing with TBS-T, the protein was characterized by detecting horseradish peroxidase activity using enhanced chemiluminescence (ECL) substrate via chemiluminescence imager (PXi, Syngene, Frederick, MD, USA). The *β*-actin cellular protein expression was detected from the same soluble proteins generated from HT29/HSV-*tk* and parental HT29 cells as internal loading control by using a mouse *β*-actin monoclonal antibody as a primary antibody (1:500 dilution) and mouse IgG_K_ binding protein-HRP antibody as a secondary antibody (1:1000 dilution).

### 3.10. Development of GCV–PCL–Chitosan/HSV-tk Nanovectors

For one-step delivery of gene and prodrug to cells, GCV–PCL–chitosan/HSV-*tk* nanovectors (i.e., GCV–PCL–chitosan micelles complexed with HSV-*tk* gene plasmid) were made. GCV–PCL–chitosan micelles with various concentrations (25, 75, and 250 µg/mL) were prepared. To 1 mL aliquots of micelle solution, various amounts (1.5, 2.0, 3.0, and 4.5 µg) of HSV-*tk* gene encoding plasmid was added to form GCV–PCL–chitosan/HSV-*tk* nanovectors. The solution was vortexed briefly and then allowed to incubate at room temperature for 30 min prior to DLS and zeta potential analysis.

### 3.11. Cytotoxicity Studies

HT29 colorectal cells were inoculated in 24-well plates containing 0.5 mL Dulbecco’s modified Eagle medium (DMEM), supplemented with 10% fetal bovine serum (FBS) and 1% penicillin-streptomycin, and then cultured at a 37 °C incubator balanced with 5% CO_2_ for overnight. For the endocytic uptake study, the cells were treated with GCV–PCL–chitosan polymeric micelles loaded with hydrophobic Nile Red. After 6 h culture, the medium was disposed and the cells were stained with DAPI and observed under a fluorescent microscope. For the two-step GEDPT approach, parental HT29 cells and HSV-*tk* gene transfected HT29 cells (HT29/HSV-*tk*) were treated with various concentrations (0.025, 0.075, and 0.25 mg/mL) of GCV–PCL–chitosan polymeric prodrug micelle solution. For the one-step GDEPT approach, HT29 parental cells were treated with GCV–PCL–chitosan/HSV-*tk* nanovectors. After 72 h incubation, cell viability was examined by the MTT assay. 200 µL of sterile MTT solution (4 mg/mL) was loaded into the culture wells for 4 h. After removing the medium, 300 µL of dimethyl sulfoxide was added to dissolve insoluble purple formazan crystals deposited in mitochondria. The absorbance at 590 nm was obtained with a microplate reader (SpectraMax M2e, Molecular Devices, Sunnyvale, CA, USA) and results were recorded as viability percentage, calculated against the control group without micellar treatment.

## 4. Conclusions

To expand the range of tumors susceptible to cytotoxic drugs converted from enzyme-catalyzed prodrug, gene-directed enzyme prodrug therapy (GDEPT) has been developed. However, the delivery of prodrug and gene was operated via a two-step process. Our study demonstrates that cationic ganciclovir-embedded polycaprolactone-chitosan polymeric micelles formed polyplexes with negatively charged HSV-*tk* plasmids could consolidate the two-step GDEPT into a one-step process. Our results indicate that GCV–PCL–chitosan polymeric prodrug micelles/HSV-*tk* nanovectors could eliminate up to 50% of HT-29 colorectal cancer cells.

## Figures and Tables

**Table 1:** Characterization of GCV–PCL–chitosan ^a^.

Sample	M_w_ (Da)	M_n_ (Da)	Polydispersity (M_w_/M_n_)
GCV–PCL	12,996	11,454	1.13
GCV–PCL–chitosan	20,354	17,231	1.18

^a^ Measured by GPC.

**Table 2:** The size and charge of GCV–PCL–Chitosan/HSV-*tk* DNA nanovectors.

Amount of DNA (µg)	Size ± SD (nm)	Zeta Potential ± SD (mV)
0	93.4 ± 2.5	38.5 ± 2.7
1.5	92.4 ± 0.9	37.9 ± 5.3
2.0	92.4 ± 1.2	37.5 ± 1.8
3.0	92.8 ± 0.7	30.1 ± 3.1
4.5	128.4 ± 1.5	28.2 ± 0.9

SD = standard deviation.

## Data Availability

The data presented in this study are available on request from the corresponding author.
